# An interactive model for the assessment of the economic costs and benefits of different rapid diagnostic tests for malaria

**DOI:** 10.1186/1475-2875-7-21

**Published:** 2008-01-28

**Authors:** Yoel Lubell, Heidi Hopkins, Christopher JM Whitty, Sarah G Staedke, Anne Mills

**Affiliations:** 1 Department of Public Health and Policy, LSHTM, London, UK; 2 University of California, San Francisco, USA and Uganda Malaria Surveillance Project, Kampala, Uganda; 3 Department of Infectious and Tropical Diseases, LSHTM, London, UK

## Abstract

**Background:**

Rapid diagnostic tests (RDTs) for malaria are increasingly being considered for routine use in Africa. However, many RDTs are available and selecting the ideal test for a particular setting is challenging. The appropriateness of RDT choice depends in part on patient population and epidemiological setting, and on decision makers' priorities. The model presented (available online) can be used by decision makers to evaluate alternative RDTs and assess the circumstances under which their use is justified on economic grounds.

**Methods:**

An interactive model based on a decision-tree structure and a cost-benefit framework was designed to compare different diagnostic strategies. Variables included in the model can be modified by users, including RDT and treatment costs, test accuracies (sensitivity and specificity), probabilities for developing severe illness, case-fatality rates, and clinician response to negative test results. To illustrate how the model can be used, a comparison is made of presumptive treatment with two available RDTs, one detecting histidine-rich protein-2 (HRP2) and one detecting Plasmodium lactate dehydrogenase (pLDH). Data inputs were obtained from a study comparing the RDTs at seven sites in Uganda.

**Results:**

Applying the model in the illustrative Ugandan context demonstrates that if only direct expenditures are considered, the pLDH test is the preferred option for adult patients except in high transmission settings, while young children are best treated presumptively in all settings. When health outcomes are considered, the HRP2 test gains an advantage in almost all settings and for all age groups. Introducing possible adverse consequences of using an antimalarial into the analysis, such as adverse drug reactions, or the development of resistance, considerably strengthens the case for using RDTs. When the model is adjusted to account for less than complete adherence to test results, the efficiency of using RDTs drops sharply.

**Conclusion:**

Model output demonstrates that which test is preferable varies by location, depending on factors such as malaria transmission intensity and the costs and accuracies of the RDTs under consideration. Despite the uncertainties and complexities involved, adaptable models such as the one presented here can serve as a practical tool to assist policy makers in efficient deployment of new technologies.

## Background

### The role of RDTs and decisions in their implementation

In sub-Saharan Africa, management of febrile patients is typically characterized by over-prescription of antimalarial drugs [1-4], as clinicians often do not have access to, or do not request, laboratory testing before prescribing antimalarials [4,5]. Such practices were accepted, and even encouraged, when older, more affordable antimalarials such as chloroquine and sulphadoxine-pyrimethamine were effective. However, now that parasite resistance necessitates the introduction of new regimens such as artemisinin combination therapies (ACTs) [6-9], the strategy of presumptive treatment has become more problematic, as the new drugs are significantly more expensive and their safety profiles are not fully characterized. Use of rapid diagnostic tests (RDTs) to guide antimalarial therapy is increasingly advocated as a potentially safe and cost-effective strategy for fever case management [10-13].

With an increasingly large number of RDTs available on the market, decision-makers must consider a number of factors in determining which diagnostic test is likely to be most appropriate in a particular context. Some of these relate to qualities of the RDT itself, such as target antigen, sensitivity, specificity, shelf-life, heat sensitivity and cost. Other factors relate to the demographic and epidemiological circumstances of areas where the tests are to be deployed. Some data are available, for example from field studies of RDT accuracy in various settings, but data are lacking for other critical parameters that are likely to influence the overall costs and benefits of implementing RDTs. Even where data are available, many of these factors vary even within a single country or region, presenting a complicated picture to decision-makers.

The availability, performance and prices of diagnostic tests and treatments can vary widely over time and location, as do transmission intensity and host immunity. It is unlikely therefore that any RDT would maintain its advantage indefinitely or across all endemic areas. Similarly, economic evaluations of an RDT carried out in one setting may not be relevant in others, or lose their validity within a relatively short time as epidemiological patterns and the characteristics of competitor tests changes. For these reasons, policy makers might benefit from decision aids that incorporate available data and parameter estimates for factors that are variable, to provide up-to-date recommendations relevant to their circumstances.

### Factors for consideration in choice of RDT

The presumptive treatment of fever episodes as malaria results in significant overuse of antimalarials and delays diagnosis of other illnesses [14-16]. Therefore, an important potential gain from introducing a new diagnostic test is in reducing the proportion of febrile patients who receive unnecessary antimalarial treatment. This safely reduces the cost of giving unnecessary antimalarials, and may help to avert morbidity associated with untreated non-malaria illness. An ideal RDT should therefore have high specificity to avoid false-positive results that would prompt unnecessary antimalarial treatment. At the same time, it is critical that an RDT must have high sensitivity to ensure that true cases of malaria are detected and treated appropriately.

In reality, improved sensitivity often comes at the expense of reduced specificity, and vice versa; however, it is difficult to weigh the implications of this trade-off for an individual patient or for public health, as they are often not directly comparable [17]. Mistakenly diagnosing a patient as uninfected (a false negative) may have more serious clinical consequences than mistakenly diagnosing a patient as infected (a false positive), but this will not always be true. However withholding antibiotics from a malaria test-negative individual because of an assumption the illness is due to malaria may lead to treatable bacterial disease progressing to become potentially life-threatening. Extensive overuse of antimalarials is also likely to come at a considerable cost over the longer term due to increased drug pressure leading to possible development of drug resistant parasite strains [2].

The trade-off in sensitivity and specificity is apparent in the reported accuracies of the two main classes of RDTs which currently appear most suitable for clinical use, detecting either histidine-rich protein-2 (HRP2) or Plasmodium lactate dehydrogenase (pLDH). HRP2 based assays have shown good sensitivity in a variety of field settings, and are increasingly advocated where reliable microscopy is not available [11,18]. Their potential disadvantage however, is persistence of detectable circulating antigen for up to several weeks after parasites have been eradicated [19-21], which may limit the usefulness of HRP2-based assays in areas of high malaria transmission. pLDH-based RDTs appear to be less sensitive but are more specific than HRP2 ones, as the antigen is rapidly cleared from the bloodstream [22-24]. HRP2- and pLDH-based tests also differ in the parasite species they detect: the HRP2 test detects only *Plasmodium falciparum*, while the pLDH test detects all four human malaria species.

For two main reasons, evaluations of diagnostic tests should also account for important differences in malaria epidemiology and population characteristics. Firstly, transmission intensity determines prevalence of parasitaemia and therefore, the probability of a test result being correct (the positive and negative predictive values). In many areas parasite prevalence varies through the year due to seasonal fluctuations in transmission intensity. Secondly, in high transmission areas the population develops partial immunity with age [25]. An adult in a high transmission area, for instance, is more likely to be parasitaemic, but much less likely to develop severe malaria. A child in a low transmission area, on the other hand, is less likely to be parasitaemic but more likely to develop severe malaria once infected. The implications and benefit of using an RDT in each setting therefore differ [10,26,27].

Alongside the benefits of correct use of antimalarials, as for any medication, there are also possible negative consequences. The "harm of treatment" for an antimalarial or antibiotic includes the potential for drug toxicity, the contribution to the development of parasite (or bacterial) resistance, and the cost of the use of scarce resources [11]. Evaluations that account for these consequences can provide more comprehensive estimates of the real costs and benefits of various diagnostic strategies than those focusing only on immediate implications for management of a single fever episode.

This paper presents a model designed to incorporate local and current data and parameter estimates to assist stakeholders in identifying the most efficient tests and case management strategies. The aim was to develop a model that can be adapted to varied settings and RDTs, rather than to determine RDT cost-effectiveness in a generalized manner. The model expands on other available models, including one that compares the use of RDTs with microscopy and presumptive treatment [27], and data that demonstrate the importance of clinician response to test results [28]. The model presented here broadens the range of factors included in the analysis and also provides users with greater ability to explore policy options.

Use of the model is demonstrated here by comparing presumptive treatment with two RDTs proposed for deployment in peripheral outpatient departments in Uganda.

## Methods

The model was designed to amalgamate the costs and consequences of diagnosing and treating patients according to results of either of the proposed RDTs or by presumptive treatment. The model was then populated with sample data from field studies in Uganda to illustrate its function and limitations, and to demonstrate the effect of changes in each variable on model output. While the data and output are relevant to these particular settings, they are presented here only for the purpose of illustrating use of the model, not as generalizable policy recommendations. Decision makers will want to review model parameters and modify these to their own circumstances where appropriate.

### The model structure

The model is based on a decision-tree structure and cost-benefit framework, incorporating consequences of diagnosis and treatment to estimate the total costs, representing both expenditure and outcomes, for each of the tests. Strategies compared in the model include case management based on the results of two diagnostic tests, or presumptive treatment without a confirmed diagnosis.

Monetary values were assigned to consequences of diagnosis and treatment, incorporating costs of tests, medications and inpatient care, and a cost representing the value of life years lost due to incorrect diagnosis and treatment. As both costs and consequences of the different strategies are expressed in monetary terms, these are differentiated in the text by referring to either 'direct costs' to describe financial expenditures alone, or to 'total costs' where both financial expenditures and consequences in terms of value of life years lost are included. The option that incurs the lowest total cost is considered the most efficient.

### Assignment of monetary values to health outcomes

The probability of death occurring was determined using estimates for the likelihood of untreated malaria and other febrile illnesses becoming severe, and subsequent case fatality rates. These were determined using expert opinion due to lack of clinical data. Different probabilities were assigned to different age groups and transmission intensities, as detailed in Table 1.

**Table 1:** Transition probabilities used in the model. NMFI – Non malarial febrile illness. CFR – Case fatality rate.

**Probability untreated malaria becomes severe**	**Age group**			**Source**
**Transmission intensity**	**Under 5**	**5 to 10**	**10+**	

Low	0.075	0.05	0.01	[25, 32, 41]; supplemented by expert opinion (Chris Whitty, Hugh Reyburn)
Medium	0.075	0.01	0.004	
High	0.075	0.01	0.0015	
**CFR severe malaria**	0.2	0.2	0.2	
**Probability untreated NMFI becomes severe**	0.01	0.005	0.010	
**CFR NMFI**	0.1	0.20	0.30	

The value assigned to a year of life lost (YLL) was initially set at $150, based on guidance from WHO for a threshold below which averting the loss of a disability adjusted life year (DALY) is considered cost-effective [29]. An alternative method used to value a YLL is to multiply per capita gross national income (GNI) by three, as discussed in a report by the WHO Commission on Macroeconomics and Health [30,31]. Results are presented for both values.

Costs were also assigned to the potential negative consequences of using antimalarials and antibiotics, or the 'harm of treatment'. The initial input used was the only current available estimate for the harm of treatment incurred by the use of antimalarials, that for every 200 treatments currently given, one life will be lost at some time in the future due to allergic reactions, development of drug resistance, use of scarce resources, and inappropriate treatment of other illnesses [11]. The baseline estimate for the harm of treatment with antibiotics was set equal to that of antimalarials. Recognizing the uncertainty around these estimates, the effects of variation in these values can also be explored by the user.

**Figure 1:**
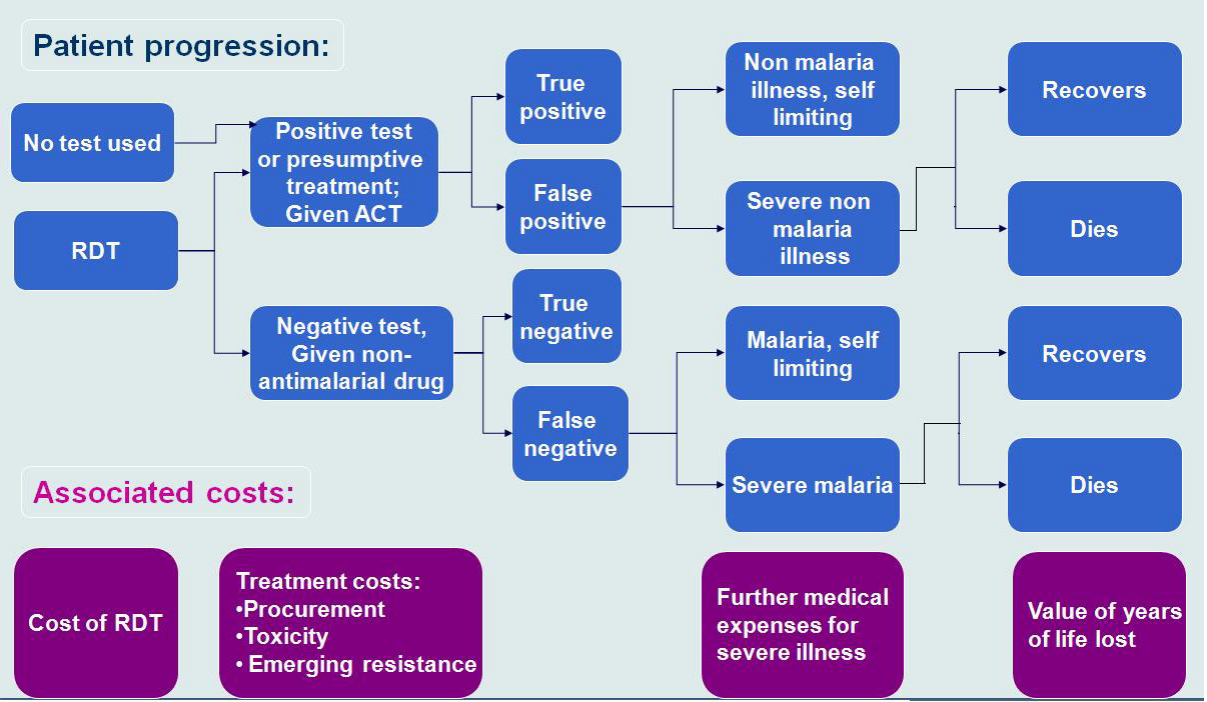
**Decision tree structure**. Patient progression and associated costs in a decision tree simulating the management of febrile patients. CFR – case fatality rate.

The model also accommodates the possibility that clinicians might continue to prescribe antimalarials in the face of negative test results. The values used in generating the results presented in this paper appear in Table 2. Figure 1 illustrates the possible outcomes and related costs for each diagnostic approach.

**Table 2:** Initial parameter estimates used in the model

**Parameter**	**Base estimate and alternatives**	**Source**
**Costs:**		
ACT	$1.8 (adult dose)	Uganda MoH
Antibiotic	$0.4 (adult dose)	Primary data – Joint Medical Store
pLDH RDT	$.51	Manufacturer
HRP2 RDT	$.55	Manufacturer
Harm of treatment with ACT (or antibiotic)	Every 200/2000 ACT doses currently used result in the loss of one statistical life	[11]
Inpatient care severe malaria	$12	Primary data, Kisiizi Hospital
Inpatient care severe NMFI	$20	
**Accuracies:**		
pLDH sensitivity	77.1%	Primary trial data
pLDH specificity	98.4%	Primary trial data
HRP2 sensitivity	98.8%	Primary trial data
HRP2 specificity	87.0%	Primary trial data
**Illness progression probabilities:**		
Adherence	Full adherence -100%	Variable in model
Year of Life Lost (YLL)	$150, $840	[29-31]

### The model interface

**Figure 2:**
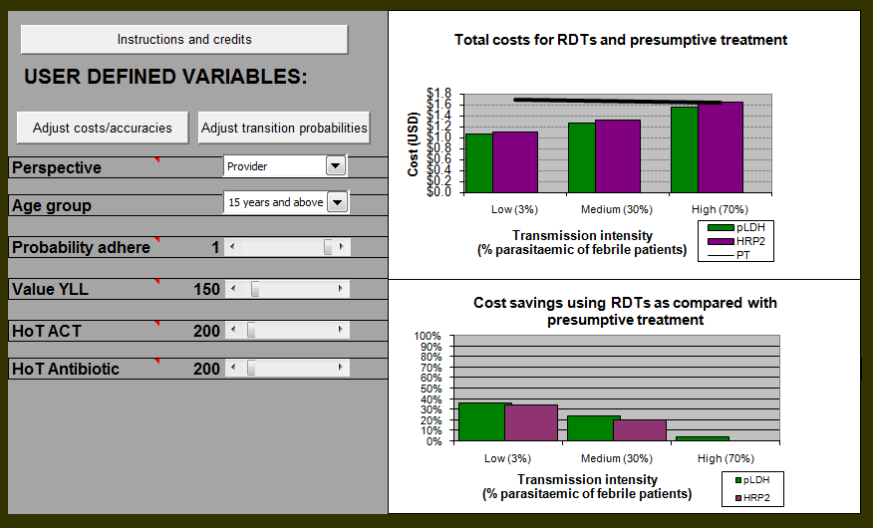
**The user interface**. RDT and drug costs, and test accuracies can be accessed and changed using the assigned button on the left hand panel, as can the probabilities of developing severe illness and case fatality rates. Other parameters can be adjusted or excluded using scroll bars on the left panel. The updated results appear on the right hand side. The bars in the top right panel show the total cost for each RDT by varying levels of prevalence, and the trendline depicts the costs for presumptive treatment. The bottom graph displays the relative cost savings for each of the RDTs using presumptive treatment as a baseline, again by prevalence level. PT – presumptive treatment

The user interface allows for variation of input parameters, making the model adaptable to different antimalarial and RDT costs, and to different test accuracies (Figure 2). The interface also enables the user to vary estimates for key parameters with strong elements of uncertainty. These include the probability of developing severe illness by age and transmission intensity, the case fatality rates for malaria and non-malarial febrile illness, and the probability that clinicians adhere to test results.

The user can also choose the perspective of the analysis. Taking the provider financial perspective considers only direct costs of tests and treatment. Alternatively the value of years of life lost to patients due to incorrect diagnosis can be added to the analysis and varied to capture immediate health benefits for the patients. Finally, a societal perspective can be taken, with the incorporation of the harm of treatment factor.

The model output is displayed on two graphs reflecting the difference in total costs in both absolute and relative terms, across three transmission intensities, defined by prevalence of parasitaemia amongst febrile patients [28,32]. Low transmission is characterized by a prevalence of 3% parasitaemia, 30% in medium, and 70% in high. This allows users to view the most appropriate RDT with respect to regional and seasonal variation in transmission intensity. In the top panel of Figure 2, the trendline represents the total cost in US$ of presumptive treatment in absolute terms, while each set of bars is the cost for either RDT at each transmission intensity. Where the bars fall below the trendline, use of the RDT would, therefore, be more efficient than presumptive treatment. In the lower panel the results are displayed in relative terms, using presumptive treatment as the baseline, so the bars represent the percentage by which RDTs are more efficient than presumptive treatment. Both graphs are included as in some cases the difference in relative terms might seem small, but is large in absolute terms, and vice versa.

The model was designed using Microsoft Excel^® ^2002 and macros were written with Microsoft Visual Basic^® ^6.3.

#### RDTs under consideration

The two RDTs evaluated for illustrative purposes in this report are Paracheck^® ^(Orchid Biochemicals Systems Goa, India) detecting HRP2 antigens, and Parabank^® ^(Zephyr Biomedicals, Goa, India) detecting pLDH antigens. The results are not generalisable to other settings for either the specific tests or the class of tests; these are an illustration of the uses of the model for policymakers from a particular setting. The data on RDT accuracy were obtained in clinical evaluations at sites with varied malaria epidemiology around Uganda, as has been described elsewhere [33]. Briefly, at each site, 1,000 consecutive outpatients referred to the laboratory for malaria screening, according to the usual standard of care at the health centres, were studied. For all samples where an RDT result was discordant with the microscopy result, polymerase chain reaction (PCR) was performed to confirm the presence or absence of parasitaemia. Sensitivity and specificity for each RDT were then calculated using PCR-corrected expert microscopy as the gold standard. Malaria prevalence in symptomatic patients at each site was defined as the proportion of parasitaemic patients according to the gold standard, and was assumed to be an indication of transmission intensity [34].

#### RDT and treatment costs

Treatment was assumed to be with artemether-lumefantrine (Coartem^®^), Uganda's recommended first-line treatment for uncomplicated malaria. Treatment costs for ACTs and antibiotics were determined by patient age: the cost of a dose for a child under five years of age was assumed to be one third of that for an adult, while for children aged 5 to 14 years the value used was two thirds of an adult dose. This corresponds with the figures provided by the Uganda Ministry of Health (Dr Fred Kato, Malaria Control Programme, personal communication, 27 April 2007). RDT costs were obtained from the manufacturer and incorporated an additional 15% on top of purchase price for transport and wastage [10]. Direct costs of inpatient care for patients with severe illness were estimated using primary data from Kisiizi Hospital in southwest Uganda.

## Results

To demonstrate the structure and functions of the model, sample outputs are presented in a step-wise fashion, beginning with direct diagnostic and treatment costs alone. This is followed by the inclusion of patient health outcomes using the estimated values for YLL. The impact of varying levels of prescriber adherence to RDT results is then explored. The model output becomes fully comprehensive when finally the estimates of harm of treatment are incorporated. The sensitivity of the results to changes in each of the input parameters is presented as they are introduced. For simplicity, only absolute and not relative costs for each strategy are presented.

### Direct cost comparison

**Figure 3:**
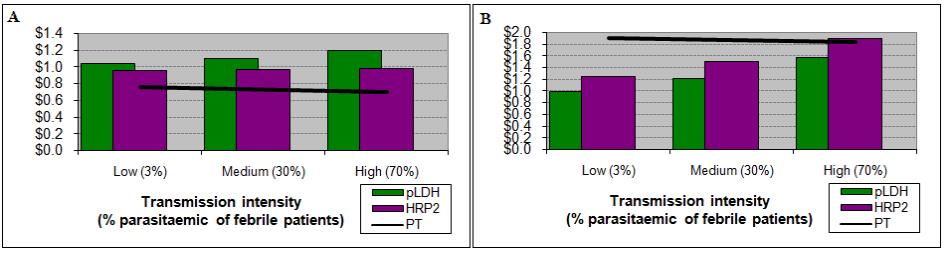
**Results with direct costs alone**. Costs of diagnosis and treatment for children under 5 (left) and for adults (right). For children the consideration of direct costs alone implies that presumptive treatment is the preferred option across all prevalences. For adults the RDT bars remain below the presumptive treatment trendline, indicating that the use of RDTs is less costly than presumptive treatment. PT – presumptive treatment

For patients under five years of age the current cost of ACT is only marginally more expensive ($0.02) than either RDT. Therefore, if health outcomes are excluded from the analysis, presumptive treatment is the preferred option across almost all settings for this age group (Figure 3a). For patients aged five to 14 years, use of either RDT is less costly in low and medium transmission intensities, and roughly equal in the high one (result not shown). For adults both RDTs, and particularly the pLDH test, are less costly in all settings (Figure 3b).

Considering only direct expenditure excludes important factors. For example, the advantage of the pLDH test is explained in part by its lower sensitivity, resulting in fewer antimalarials being prescribed for true cases of malaria and, therefore, a lower expenditure. To capture the full cost of these untreated malaria cases in the model, the value of years of life lost due to incorrect diagnosis and treatment must be incorporated.

### Introducing the value of YLL

**Figure 4:**
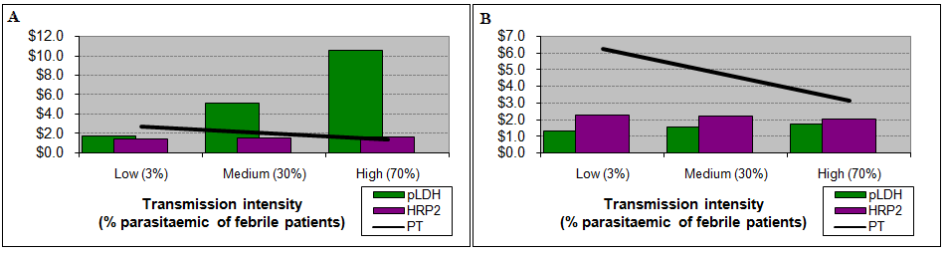
**Results incorporating the value of health outcomes**. Costs for children under 5 years (left) and adults (right), incorporating the value of life years lost. For young children presumptive treatment maintains a slight advantage over the HRP2 test, while the pLDH test would incur significantly higher costs, particularly at higher transmission intensity. For adults either test would be slightly more efficient than presumptive treatment, with a slight advantage to the HRP2 test up to very high prevalences. PT – presumptive treatment

Initially, a baseline value of $150 for a YLL was used. For patients under five years of age, the introduction of this value provides both RDTs with an advantage at the low transmission setting; at higher transmission intensities this advantage is maintained by the HRP2 test, although decreasingly so as transmission increases. Use of the pLDH test is least efficient, particularly in high-transmission areas, due primarily to its lower sensitivity and consequent failure to diagnose and treat true malaria (Figure 4a). When the value of a YLL is increased to $840 (three times Ugandan GNI per capita) [35], there is a modest further increase in the benefit of using the HRP2 test.

For older children, the HRP2 test has a small advantage over the pLDH test across all three settings, with the pLDH test being more costly than presumptive treatment at the high transmission intensity. For adults both RDTs are more efficient than presumptive treatment across all settings (Figure 4b), with substantial cost savings at sites with lower transmission.

### Adherence

Results so far assume that clinicians prescribe treatment that are consistent with test results in prescribing treatments. However, consistent responses cannot be assumed given evidence from many areas showing that antimalarials are often prescribed even if test results are negative, and the degree of consistency affects comparisons [4,26]. For children aged five to 14 years for instance, the advantage gained by using the HRP2 test is lost once adherence falls below approximately 65%, and presumptive treatment becomes the preferred option.

### Harm of treatment

**Figure 5:**
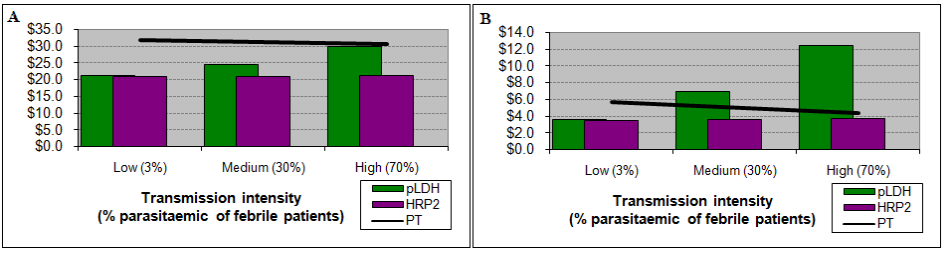
**Results with the harm of treatment included**. Total costs for children with a high (left) and low (right) estimate of harm of treatment associated with provision of antimalarials and antibiotics. Even with a very conservative estimate of the potential harm of treatment, the HRP2 test maintains an advantage across all prevalences. PT – presumptive treatment

When the harm of treatment associated with over-prescription of antimalarials is included, results change considerably in favour of either RDT. The baseline estimate implies that for every 200 ACTs given, one statistical life is lost in the future [11]. Figure 5a demonstrates that for children under five years, where previously presumptive treatment was the preferred option, when the harm of treatment is added to the analysis the use of RDTs is substantially more efficient across all settings. Recognizing the uncertainty around this value, a second value of 2,000 was arbitrarily chosen to observe the sensitivity of results to a lower estimate of harm of treatment. Even with much lower estimate of harm of treatment, the HRP2 test remained the most efficient choice (Figure 5b). At the medium transmission intensity, the harm of treatment value would have to be above 7,000 (i.e. prescription of 7,000 antimalarials equates to one statistical death) before presumptive treatment again becomes the more efficient option.

For older patients results are similar, with almost no difference between the two RDTs – both being 30% to 50% more efficient than presumptive treatment at the lower transmission intensities. This advantage was maintained by the HRP2 test in areas of high transmission as well. This result was robust to reduction in the value of the harm of treatment.

## Discussion

### Adaptable economic models as decision support tools

ACTs are a valuable resource and use of RDTs to target therapy is likely to be preferable to presumptive treatment in certain settings. A variety of RDTs are available, each with potential advantages and disadvantages, and deciding on the appropriate diagnostic approach for a given setting can be challenging. This paper presents a model which can compare different rapid diagnostic tests with one another and presumptive treatment. Policymakers can vary the parameters depending on local conditions, new data, and their own opinions where data are not available. The model demonstrates that which diagnostic strategy or test is likely to be cost-effective depends on setting, and perspective.

This model aims to be useful to stakeholders and decision makers in a number of ways. Firstly, by demonstrating the variation in performance by patient age and transmission intensity, policies may be better targeted to the local environments and patient populations. While it may not be feasible in all cases to implement policies that vary by region or population, as this may add costs and complexities to the implementation process, considering the possible variation will provide more accurate and nuanced data to inform the development of national strategies. Secondly, the interactive nature of the model allows policymakers to select which input parameters are relevant, and to use values that reflect the local settings. Thirdly, the model can be used to identify influential parameters for which values are uncertain, and to indicate the need for investing in further research to derive more accurate estimates.

Models can appear to make highly complex policy dilemmas overly simplistic, but despite all the uncertainties and complexities, decisions regarding the use of RDTs are being made, often using little more than intuitive inclination in the absence of better data. Models such as this assist in seeking to synthesise a large array of parameters that should all enter the decision making process.

### Decision and policy implications in the Ugandan context

Using the RDT accuracy data available for Uganda as an illustration, the model suggests that at current RDT and ACT prices, use of the illustrative HRP2 RDT would be appropriate across most endemic settings and patient age groups. However, the results of the model depend to a great extent on whether factors such as the harm of treatment and the probability of clinicians adhering to results are included in the analysis.

If the model is set to exclude the harm of treatment, as ACTs drop in price, presumptive treatment becomes justified for younger children, and the advantage of RDTs for older patients is greatly reduced. Results of the model highlight to policy makers the importance of encouraging clinicians to adhere to negative test results, if RDTs are to be an efficient use of resources.

## Limitations

For some parameters in this model, such as harm of treatment, only rough estimates are available, and in many settings, local data for other parameters affecting RDT choice are unavailable. However, use of reasonable estimates and exploration of the effects of their variation in the model may provide a useful guide for decisions on RDT implementation. For purposes of illustration the model is initially populated here with current best estimates, as is the case in standard evaluations. With use of the model, users may modify these with local data where available, to tailor results as far as is possible to their own circumstances.

Two factors that were not accounted for in the model are drug efficacy, and the quality of life during illness or due to neurological sequelae. These were excluded assuming that they would have equal impact on all arms, and therefore would not alter decision recommendations. Shelf life of RDTs and stability at high temperatures are two operational factors that cannot be modelled reliably given current knowledge, but which may need to be taken into account in local settings in addition to predictive value and cost-effectiveness.

The difficulties surrounding the assignment of monetary values for years of life lost has been discussed extensively in the literature [30,36-39]. The values used in this analysis were derived from two commonly used methods – one representing a threshold for willingness to pay for a DALY averted derived by the WHO [29], and the other reflecting productivity costs by using a multiple of GNI [30,31]. In this analysis these measures have been used to value YLLs, which as opposed to DALYs do not account for a quality of life dimension. This was considered acceptable as in the context of malaria, the quality of life component is assumed to be of marginal importance in comparison to the loss of life years [40], so the two measures are almost equivalent.

The parameter surrounded with most uncertainty is the potential harm of treatment with antimalarials (or any other medication). Quantifying this requires challenging assessments such as the probability of toxicity, and the relationship of quantities of ACTs used to development of resistance, which can make the estimates appear rather arbitrary. The baseline estimate used is the only one currently available in the literature. Given this uncertainty, this parameter was varied by one order of magnitude to test its robustness, followed by a threshold analysis to determine the point where presumptive treatment again becomes more efficient.

Despite the difficulties in estimating this parameter, it is important that whatever estimates are available be accounted for in a decision model. Excluding a value for potential harm of treatment essentially can equate to saying the long term costs associated with widespread use of antibiotics or antimalarials are zero. The model allows the user to observe how changes in these values influence decision recommendations. The assignment of an equal cost to antibiotics was done on expert opinion, although users are encouraged to question this and where appropriate enter their own estimates in the model.

## Conclusion

This paper presents a model that explores important parameters influencing RDT costs and benefits, that can be used by decision makers to evaluate alternative RDTs and assess the circumstances under which their use may be justified on economic grounds. It demonstrates the importance of the epidemiological setting in determining which test is most appropriate. The model is suitable for use with local data concerning test accuracies and costs of diagnostics and treatments, and allows policy makers and other stakeholders to use their own estimates for a variety of other parameters. Sample data are used to demonstrate how the model can be used to provide recommendations relevant to RDT implementation in the Ugandan context.

The question of which diagnostic approach is most cost-effective does not have a single correct answer. This paper demonstrates how in a diverse and rapidly evolving environment, adaptable and responsive models can offer guidance to encourage the most efficient deployment of new technologies.

## Model availability and requirements

Project name: RDT Decision Support Model

Project home page: 

Operating system(s): All systems supporting Microsoft Office^® ^software with Macros enabled in Excel

Programming language: Microsoft Excel ^® ^2002 and Microsoft Visual Basic^® ^6.3

## Authors' contributions

YL designed the model; HH and YL wrote the initial manuscript draft; all authors contributed equally to the final content.
